# Analysis of Clinical Characteristics of Tuberculosis Patients with Dementia in Gyeongsangbuk-do, Republic of Korea

**DOI:** 10.3390/jcm13051215

**Published:** 2024-02-21

**Authors:** YouKyoung Kim, Seok-Ju Yoo, NaYoung Hong, Kwan Lee

**Affiliations:** 1 Gyeongsangbuk-do Infectious Disease Control Division, Andong-si 36759, Gyeongsangbuk-do, Republic of Korea; dbruddl9975@korea.kr; 2 Department of Preventive Medicine, College of Medicine, Dongguk University, Gyeongju 38066, Gyeongsangbuk-do, Republic of Korea; kwaniya@dongguk.ac.kr; 3 Gyeongsangbuk-do Center for Infectious Diseases Control & Prevention, Andong-si 36759, Gyeongsangbuk-do, Republic of Korea; gbcidc5@gbcidc.or.kr

**Keywords:** tuberculosis, dementia, tuberculosis patients with dementia, cure rate, mortality rate, people over 50, aging societies, chronic disease, geriatric diseases

## Abstract

(1) Background: Among Korean research papers there have been studies on the correlation between tuberculosis-hypertension and diabetes and the correlation between dementia-hypertension and diabetes, but there were no analysis data specifically on tuberculosis and dementia. (2) Methods: A total of 2992 tuberculosis patients in the Gyeongbuk region were analyzed through a final analysis of integrated disease and health management system data collected from 2021 to 2022. In this selection, patients with tuberculosis under 50 years of age and 368 people diagnosed with tuberculosis were excluded. (3) Results: From 2021 to 2022, among the 2992 tuberculosis patients in Gyeongsangbuk-do aged 50 or older, 2722 (91.0%) belonged to the general tuberculosis patient group, while 270 (9.0%) belonged to the dementia–tuberculosis patient group. The average age in the dementia–tuberculosis group was 81.4 years, significantly higher than the general group’s average of 75.7 years. Within the dementia–tuberculosis patient group, 235 patients (87.0%) had underlying medical conditions in addition to dementia and tuberculosis. The tuberculosis treatment cure rate was 56.3% (1477 patients) in the general group and 38.9% (105 patients) in the dementia–tuberculosis patient group. (4) Conclusions: The cure rate was notably higher in the general group. Similarly, the mortality rate (deaths due to tuberculosis) was significantly higher in the dementia–tuberculosis patient group (7.0%, 19 patients) compared to the normal group (3.0%, 81 patients). The mortality rate in the dementia group was more than twice that of the general group.

## 1. Introduction

The World Health Organization (WHO) highlights that the current human population is the first generation in history with an average life expectancy of 60 years. In 2015, there were an estimated 900 million people over 60 (12% of the total population). This number is projected to double to 2 billion by 2050, representing a 21% increase in the overall population [1].

This demographic shift poses a significant challenge. While material abundance and technological advancements have extended average human lifespans, the crucial task of maintaining health during these extended years has become significantly more demanding [2]. Recognizing this, healthcare policies are increasingly focusing on the prevention and management of chronic and geriatric diseases in the elderly population. This long-term approach requires strong and diverse voices to advocate for effective solutions [3].

Tuberculosis (TB) is a frequently cited chronic disease requiring national management due to its significant public health impact. Both domestic and international efforts emphasize the importance of TB prevention and control [4]. A 2021 WHO report highlights the burden of TB globally, with an estimated 10.6 million new cases and 1.6 million deaths attributed to the disease in that year alone. TB is projected to become the second-leading cause of death from infectious diseases worldwide, surpassed only by coronavirus infection-19 (COVID-19) [5]. In Korea, TB occupies the same position, following COVID-19 (5030 deaths), with 1430 deaths in 2021 [6].

In the context of aging societies, managing geriatric diseases is crucial alongside chronic disease management. Dementia, receiving widespread societal attention, stands out as a primary concern. Globally, an estimated 55 million individuals currently live with dementia, with a projected near doubling of this number by 2030 [7,8]. Korea’s rapidly aging population reflects this trend, with a rising number of elderly individuals experiencing dementia. As of March 2022, the Central Dementia Center’s Dementia Status Report estimated approximately 880,000 dementia patients nationwide, resulting in a 10.3% dementia prevalence. Notably, dementia affects a significant portion of Korea’s elderly population (10.2%).

According to a 2022 report by the Korea Disease Control and Prevention Agency, the number of new TB cases in Korea decreased by 11.3% to 31.7 per 100,000 people compared to 35.7 per 100,000 in 2021. This marks a continuing downward trend since 2011.

Korea’s 2022 Senior Citizen Statistics reveal that the Gyeongbuk region has transitioned into a super-aging society, with a 22.8% elderly population, and the proportion of residents aged 65 or older is steadily increasing. Notably, the TB death rate in Gyeongbuk (4.3 per 100,000 people in 2022) surpassed the national average of 2.8 per 100,000 people. Moreover, 87.5% of TB deaths in the region occur among those aged 65 years or older.

Among Korean research papers, there have been studies on the correlation between tuberculosis-hypertension and diabetes and the correlation between dementia-hypertension and diabetes, but there were no analysis data specifically on tuberculosis and dementia. Motivated by these statistics, this study aims to analyze treatment outcomes of TB patients with dementia, a prevalent geriatric disease, in the Gyeongbuk region, thereby providing valuable basic data to inform TB prevention efforts.

## 2. Materials and Methods

A total of 2992 patients with TB in Gyeongsangbuk-do, Republic of Korea were included in the final analysis from the Integrated Disease and Health Management System (is.kdca.go.kr) data collected between 2021 and 2022. This selection excluded patients with tuberculosis under 50 years old and 368 patients with a change in diagnosis based on the treatment.

Patients without diagnosed dementia were designated as the “general TB patient group”, while those with dementia were classified as the “dementia-TB patient group”. The analysis included 2722 individuals in the general group and 270 in the dementia group and was analyzed retrospectively.

Age was categorized into four groups: 50–59 years, 60–69 years, 70–79 years, and 80 years or older. Underlying conditions included those diagnosed with dementia. Treatment outcomes were categorized as “cured” or “uncured” based on responses classified as cured, dead, undecided, failed, or discontinued. Patients with responses of dead, undecided, failed, or discontinued were grouped as “uncured”.

## 3. Statistical Analysis Method

Statistical analyses were conducted using SAS version 9.4 (SAS Institute Inc., Cary, NC, USA). Patient characteristics in the general and dementia–TB groups were compared using frequencies and percentages. The chi-square test assessed differences between groups. Logistic regression explored factors influencing TB treatment outcomes, with statistical significance considered at *p* < 0.05.

## 4. Result

### 4.1. Demographic Characteristics

This study included 2992 subjects: 2722 (55.6% men, 44.4% women) in the general TB patient group with an average age of 75.7 years, and 270 (43.3% men, 56.7% women) in the dementia–TB patient group with an average age of 81.4 years. While the general group had 11.2% more males than females, the opposite was true for the dementia group with 13.4% fewer males. Notably, the dementia group’s average age was 5.7 years higher than the general group’s average age. This age difference was confirmed to be significantly associated with dementia (*p* < 0.0001).

Further analysis revealed that 70.7% of patients in the general group and 87.0% in the dementia group had underlying diseases other than TB and dementia. This 16.3% increase in the dementia group was statistically significant (*p* < 0.0001).

Regarding reinfection cases, 16.5% of patients in the general group and 13.7% in the dementia group had a history of past TB infection. This difference was not statistically significant (*p* = 0.0103) (Table 1).

### 4.2. Bacteria Detection Rate

Among patients suspected of TB based on chest radiography, 37.95% (1033) belonged to the general TB group and 35.6% (96) to the dementia–TB group. Similarly, for positive sputum smear results, 33.5% (913) were in the general group and 32.6% (88) in the dementia patient group. These differences were not statistically significant.

### 4.3. Site of TB Occurrence

Out of the 2992 TB patients in 2021–2022, 70.7% (1925) in the general group and 72.6% (196) in the dementia–TB group had pulmonary TB, showing a slightly higher rate in the dementia group. Conversely, 23.0% (626) of the general group and 21.9% (59) of the dementia group had extrapulmonary TB, indicating a higher prevalence in the general group. Notably, a statistically significant correlation (*p* = 0.0127) was found between the TB site and dementia (Table 2).

### 4.4. Drug Resistance Results

Drug resistance rates showed no significant difference between the general and dementia–TB groups. In the general group, 106 patients (3.9%) exhibited drug resistance, compared to 8 patients (3.0%) in the dementia group (Table 3).

### 4.5. Treatment Results

The general TB patient group exhibited a significantly higher cure rate, with 56.3% (1477 individuals) cured compared to 38.9% (105 individuals) in the dementia–TB patient group (17.4% difference). The mortality rate also differed between the groups, with 432 patients (15.9%) dying in the general TB group and 83 (30.7%) in the dementia–TB patient group, resulting in a 14.8% mortality rate for the dementia group. Analyzing the causes of death, we found 81 deaths (3.0%) attributed to TB in the general group and 19 (7.0%) in the dementia–TB group, more than twice the rate in the dementia group. This association between dementia and higher TB-related mortality was statistically significant (*p* < 0.0001) (Table 4).

### 4.6. Final Results

Compared to the general TB patient group, the dementia–TB group had a significantly higher odds ratio of non-cure for TB, with an unadjusted value of 1.864 (95% CI 1.443–2.408). Even after adjusting for potential confounding variables like sex, age, and underlying diseases (excluding dementia itself), the odds ratio remained significantly high at 1.744 (95% CI 1.344–2.262) (Table 5).

## 5. Discussion

This study revealed concerning disparities in TB outcomes between patients with dementia and the general TB population. Individuals with dementia experienced a mortality rate due to TB more than twice that of the general group. Additionally, their treatment cure rate was 17.4% lower, highlighting the vulnerability of this specific patient population. 

While TB treatment does not require lifelong medication, achieving a complete cure is dependent on strict adherence to the prescribed regimen for a determined period. As long as patients comply, successful treatment is highly probable. However, cognitive impairment, a hallmark of dementia, can significantly hinder adherence, potentially leading to treatment failure, discontinuation, and the spread of drug-resistant bacteria [9]. This, in turn, can result in economic burdens and even death, as highlighted in previous research.

Recognizing the severity of TB, the Korean government classifies it as a Level 2 statutory infectious disease and has implemented dedicated management strategies. The “Comprehensive Tuberculosis Control Plan”, established every five years in accordance with the Tuberculosis Prevention Act, actively drives initiatives towards eradicating TB. These efforts have yielded positive results, with new TB cases and deaths declining by 53.7% and 39.5%, respectively, from 2011 to 2021. This steady decrease, averaging 4.9% annually, stems from various successful programs, including: the nationwide expansion of the private–public cooperation (Public-Private Mix, PPM) TB control project (since 2011), the national treasury support for out-of-pocket medical expenses associated with TB treatment (since 2011), and the implementation of the first comprehensive TB control program (2013–2017), followed by the second program (2018–2022), and the tuberculosis prevention and management strengthening measure (2019) [10,11,12].

The term “dementia” originates from the Latin word “dementatus”, initially meaning “out of mind”, signifying a state of mental incapacity. In modern psychiatry, dementia refers to “a condition characterized by multiple impairments in various cognitive domains without a loss of consciousness” [13]. It is a syndrome, not a specific disease, caused by chronic or progressive brain disorders. 

Dementia affects high-level cortical functions like memory, thinking, orientation, comprehension, calculation, learning, language, and judgment, while preserving consciousness. Cognitive decline is often accompanied by, or precedes, difficulties in emotional regulation, social behavior, and motivation. 

Medically, dementia is defined as a disease-causing impairment in daily and social activities due to decreased mental function (judgment, emotion, memory, calculation, etc.) and dysfunction of cells crucial for various cognitive functions [14]. The characteristics of dementia put patients at high risk for medical and social challenges. Furthermore, depression, common in dementia, can create a vicious cycle where patients lose interest in managing their physical illness and adhering to treatment, ultimately reducing treatment effectiveness [15,16]. Studies indicate that the risk of death for dementia patients is two to four times higher than for elderly individuals without dementia [17,18,19].

A 2012 survey by the Ministry of Health and Welfare estimated that there are approximately 540,000 elderly individuals with dementia in Korea. This number is projected to double every 20 years, reaching 1.27 million by 2030 and 2.71 million by 2050 [20]. Recognizing this growing social issue, the government implemented the first National Dementia Management Comprehensive Plan in 2008, followed by the third plan (2016–2020) alongside the 2012 Dementia Management Act. These efforts aim to reduce the number of elderly individuals with dementia and prepare for the associated social challenges [21].

This study confirmed a higher prevalence of underlying diseases other than TB and dementia in dementia–TB patients compared to the general TB population. However, data on the specific prevalence, cure rate, mortality, and management strategies for dementia–TB patients in Korea remain unavailable. This knowledge gap prompted our investigation into these crucial factors. 

Continuous management is essential to address comorbidities effectively while considering age-related factors. Reducing medication misuse and abuse necessitates a holistic approach to managing concurrent conditions. Achieving this requires developing a government-level system for information sharing amongst medical institutions and dementia relief centers. Furthermore, customized education, case counseling, and management specific to each dementia type are necessary for elderly and vulnerable groups with high treatment mortality rates. This approach can empower them to develop appropriate health behaviors. Additionally, sex- and age-appropriate educational programs are crucial to enhance understanding and compliance.

Long-term research is needed to assess the potential for recurrence and multidrug-resistant TB development in untreated dementia–TB patients. Notably, efforts to combat prejudice and negative perceptions about dementia and TB through accurate information campaigns can significantly improve social communication and patient support.

This study’s limitations include the inability to analyze the link between dementia severity and chest x-ray findings, clinical characteristics of TB in dementia patients, treatment course, and progression to multidrug-resistant TB. Additionally, we were unable to assess the treatment outcomes based on risk factors, education level, occupation, exercise habits, diet, and smoking behavior. Moving forward, establishing more active clinical management and prevention strategies for elderly people and patients with dementia, alongside further research on specific management methods for the dementia–TB population, are critical steps toward improving patient outcomes and reducing the associated burdens.

## 6. Conclusions

Through this study, it was confirmed that the mortality rate due to tuberculosis in tuberculosis patients with dementia was higher than that in the general tuberculosis patient group, and the cure rate of tuberculosis patients with dementia was also confirmed to be low. As a result, it served as an opportunity to re-recognize the importance of managing tuberculosis medication compliance in patients with dementia and awareness of dementia management. In the future, customized education, case counseling, and management for each type of dementia will be needed to ensure correct health behavior for the elderly and vulnerable groups with high mortality rates and to develop eye-level education programs.

## Figures and Tables

**Table 1:** Demographic characteristics of patients with tuberculosis (TB) in Gyeongbuk from 2021 to 2022 (*n* = 2992).

Variables	Tuberculosis Patients	Dementia *n* = 270	Non-Dementia *n* = 2722	*p* Value
Year of occurrence				
2021	1510 (50.47)	139 (51.48)	1371 (51.7)	
2022	1482 (49.53)	131 (48.52)	146 (48.3)	
Sex				<0.0001
Male	1631 (54.51)	117 (43.33)	1514 (55.62)	
Female	1361 (45.49)	153 (56.67)	1208 (44.38)	
Age				
Year	76.20 ± 11.40	81.39 ± 10.66	75.68 ± 11.34	0.1845
50–59	324 (10.83)	13 (4.81)	311 (11.43)	<0.0001
60–69	584 (19.52)	30 (11.11)	554 (20.35)	
70–79	677 (22.63)	51 (18.89)	626 (23.0)	
≥80	1407 (47.03)	176 (65.19)	1231 (45.22)	
Underlying disease *				<0.0001
Yes	2159 (72.16)	235 (87.04)	1924 (70.68)	
No	833 (27.84)	35 (12.96)	798 (29.32)	
Reinfection				0.2414
Yes	485 (16.21)	37 (13.7)	448 (16.46)	
No	2507 (83.79)	233 (86.3)	2.74 (83.54)	

* Dementia is excluded from underlying disease.

**Table 2:** Site of tuberculosis in dementia patients in Gyeongbuk, 2021–2022.

Disease	Total (%)	Dementia (%)	Non-Dementia (%)	*p* Value
				0.0127
Pulmonary tuberculosis	2121 (70.89)	196 (72.59)	1925 (70.72)	
Pulmonary tuberculosis + Extrapulmonary tuberculosis	185 (6.18)	14 (5.19)	171 (6.28)	
Extrapulmonary tuberculosis	685 (22.89)	59 (21.85)	626 (23.0)	
No inspection	1 (0.03)	1 (0.37)	0 (0)	

**Table 3:** Drug resistance results of tuberculosis patients in Gyeongbuk, 2021~2022.

Disease	Total (%)	Dementia (%)	Non-Dementia (%)	*p* Value
Drug Resistance (Positive (%))				
Drug-resistant tuberculosis	114 (3.81)	8 (2.96)	106 (3.89)	0.4458
Extensively drug-resistant pre-stage tuberculosis	2 (1.75)	1 (12.5)	1 (0.94)	
Multidrug-resistant tuberculosis	24 (21.05)	1 (12.5)	23 (21.7)	
Rifampin-only resistant tuberculosis	27 (23.68)	3 (37.5)	24 (22.64)	
Isoniazid-only resistant tuberculosis	61 (53.51)	3 (37.5)	58 (54.72)	

**Table 4:** Treatment results of TB patients in Gyeongbuk from 2021 to 2022.

Disease Outcome	Total (%)	Dementia (%)	Non-Dementia (%)	*p* Value
				**<0.0001**
Cured	1582 (52.87)	105 (38.89)	1477 (54.26)	
Dead	515 (17.21)	83 (30.74)	432 (15.87)	
Death from tuberculosis	100 (3.34)	19 (7.04)	81 (2.98)	
Deaths other than tuberculosis	415 (13.87)	64 (23.70)	351 (12.89)	
Undefined	831 (27.77)	80 (29.63)	751 (27.59)	
Failure	1 (0.03)	0 (0)	1 (0.04)	
Discontinued	63 (2.11)	2 (0.74)	61 (2.24)	

**Table 5:** The odds ratio for those who were not cured among TB–dementia patients.

Division	Non-Cured Patients	
	Crude OR (95% CI)	Adjusted OR (95% CI)
Non-dementia	1	1
Dementia	1.864 (1.443–2.408)	1.744 (1.344–2.262)

Cured vs. (event) people who were not completely cured (death, undecided, failure, or discontinuation). OR, odds ratio; CI, confidence interval; analyzed by multivariable logistic regression analysis after adjusting variables. Adjusted for sex, age, and underlying disease.

## Data Availability

Republic of Korea Disease Control and Prevention Agency’s Disease and Health Integrated Management System (https://is.kdca.go.kr).
